# Cellular Senescence in Idiopathic Pulmonary Fibrosis: Molecular Mechanisms, Pathogenic Networks, and Emerging Therapeutic Targets

**DOI:** 10.3390/diseases14060201

**Published:** 2026-06-04

**Authors:** Madina B. Baurzhan, Alexandr E. Gulyayev, Karlygash S. Absattarova, Sayagul A. Kairgeldina, Kuat Abzaliyev, Akmaral Izbassarova, Marzhan Lepessova, Karashash Absatarova

**Affiliations:** 1 Research Institute of Balneology and Medical Rehabilitation, Ministry of Health of the Republic of Kazakhstan, Astana 010000, Kazakhstan; madina_baurzhan@mail.ru (M.B.B.); akin@mail.ru (A.E.G.); sanborovoe@mail.kz (S.A.K.); 2 Laboratory of Drug Discovery and Development, Nazarbayev University, Astana 010000, Kazakhstan; 3 Joint-Stock Company “Karaganda Medical University”, Karaganda 100008, Kazakhstan; 4 Non-Profit Joint-Stock Company «Kazakh National Medical University Named After S.D. Asfendiyarov», Almaty 050000, Kazakhstan; aksha5@mail.ru; 5 Department of Internal Medicine, Faculty of Medicine and Healthcare, Al-Farabi Kazakh National University, Almaty 050040, Kazakhstan; abzaliev_kuat@mail.ru; 6 Department of Neurology, Kazakh-Russian Medical University, Almaty 050004, Kazakhstan; mar.izzhan@mail.ru; 7 Department of Epidemiology, Biostatistics and Evidence-Based Medicine, Faculty of Medicine and Healthcare, Al-Farabi Kazakh National University, Almaty 050040, Kazakhstan; karashash.absatarova@kaznu.kz

**Keywords:** cellular senescence, idiopathic pulmonary fibrosis, SASP, TGF-β signaling, senolytics, aging, pulmonary fibrosis

## Abstract

Idiopathic pulmonary fibrosis (IPF) is a chronic, progressive interstitial lung disease characterized by irreversible extracellular matrix deposition and high mortality, with aging representing its strongest risk factor. Increasing evidence suggests that cellular senescence is not merely a consequence of tissue injury but a central driver of disease progression. Senescent alveolar epithelial cells and fibroblasts contribute to impaired tissue repair and persistent fibrotic remodeling through the acquisition of a senescence-associated secretory phenotype (SASP), which promotes chronic inflammation and amplifies profibrotic signaling. This review provides a comprehensive synthesis of current evidence on the role of cellular senescence in IPF, focusing on key molecular mechanisms, including telomere attrition, mitochondrial dysfunction, oxidative stress, DNA damage response activation, and dysregulated transforming growth factor-β (TGF-β) signaling. A structured literature search was conducted using the PubMed, Scopus, and Web of Science databases to identify mechanistic, translational, and clinical studies related to cellular senescence in IPF. Relevant studies were selected based on conceptual relevance and scientific quality, and findings were qualitatively synthesized within a narrative-review framework. These interconnected pathways form self-reinforcing feedback loops that stabilize the senescent phenotype and sustain fibroblast activation. In addition, we critically evaluate emerging therapeutic strategies targeting senescence, including senolytic and senomorphic approaches, highlighting their potential to modify fundamental disease mechanisms rather than solely attenuating fibrotic progression. Preclinical and early clinical studies suggest that selective targeting of senescent cells may represent a promising avenue for intervention, although challenges related to specificity, safety, and biomarker development remain. Overall, this review positions cellular senescence as a central mechanistic link between aging and fibrosis and underscores its relevance as a translational target in IPF.

## 1. Introduction

Idiopathic pulmonary fibrosis (IPF) is a chronic, progressive interstitial lung disease characterized by irreversible decline in lung function and limited survival. Despite the availability of antifibrotic therapies, clinical outcomes remain suboptimal, which underscores the need for a deeper understanding of the mechanisms driving disease progression [1,2].

In recent years, increasing attention has been directed toward the contribution of aging-related processes to the development of IPF. However, IPF should not be regarded exclusively as a disease of elderly individuals. Multiple forms of alveolar epithelial injury—including environmental exposure, oxidative stress, genetic susceptibility, smoking, and repeated microinjury—may induce senescence-associated pathways and contribute to fibrotic remodeling across different clinical contexts. Among these, cellular senescence has emerged as a particularly important factor. Senescent cells are characterized by stable cell-cycle arrest, resistance to apoptosis, and the secretion of a broad spectrum of pro-inflammatory and profibrotic mediators, collectively referred to as the SASP [3,4]. While transient senescence may play a beneficial role in tissue repair, its persistent accumulation has been associated with chronic inflammation and pathological tissue remodeling [5].

The lung is especially susceptible to senescence-associated alterations. Alveolar epithelial cells, which are essential for maintaining tissue integrity and initiating repair after injury, demonstrate increased vulnerability to stress-induced senescence [6]. At the same time, fibroblasts exposed to a senescent microenvironment acquire a profibrotic phenotype, contributing to excessive extracellular matrix deposition and structural distortion of lung tissue [7,8]. These findings suggest that senescence is not merely a consequence of repeated injury but may actively contribute to disease progression.

At the molecular level, several interconnected pathways regulate the induction and maintenance of cellular senescence in IPF. These include telomere shortening, mitochondrial dysfunction, oxidative stress, dysregulated transforming growth factor-beta (TGF-β) signaling, and impaired autophagy [9,10,11]. Importantly, these mechanisms do not operate in isolation but form a complex regulatory network that sustains both the senescent phenotype and fibrotic remodeling processes.

Given these observations, targeting cellular senescence has been proposed as a promising therapeutic strategy. Experimental studies have demonstrated that senolytic agents and senescence-modulating interventions may attenuate fibrotic changes and improve tissue function [12,13]. However, significant challenges remain, including the heterogeneity of senescent cells, the timing of therapeutic intervention, and the potential risks associated with systemic elimination of senescent populations.

The aim of this review is to provide an integrated overview of the molecular mechanisms underlying cellular senescence in IPF, with a particular focus on key signaling pathways and emerging therapeutic approaches.

## 2. Materials and Methods

### 2.1. Study Design

This review was intentionally designed as a narrative review rather than a systematic review or meta-analysis because the objective was to provide an integrative conceptual synthesis of heterogeneous mechanistic, translational, and clinical evidence related to cellular senescence in IPF. Accordingly, PRISMA guidelines and formal quantitative evidence-synthesis methodologies were not applied. Narrative reviews are appropriate for integrating findings from heterogeneous experimental, translational, and clinical studies and for providing conceptual frameworks in rapidly evolving research fields.

### 2.2. Literature Search Strategy

A structured literature search was performed using three major electronic databases: PubMed, Scopus, and Web of Science. The search covered publications available up to January 2026 (Supplementary Materials).

The search strategy combined Medical Subject Headings (MeSH) terms and free-text keywords, including: “idiopathic pulmonary fibrosis”, “cellular senescence”, “senescence-associated secretory phenotype”, “telomere dysfunction”, “mitochondrial dysfunction”, “oxidative stress”, and “senolytics”. Boolean operators (AND, OR) were used to optimize search sensitivity and specificity.

To ensure comprehensive coverage, additional relevant publications were identified through manual screening of reference lists of selected articles.

### 2.3. Eligibility Criteria

Publications were considered eligible if they met the following criteria:

(i) Peer-reviewed articles published in English;
(ii) Studies addressing cellular senescence and/or its role in IPF;
(iii) Original research articles, reviews, or clinical studies providing mechanistic or therapeutic insights.

Studies were excluded if they:

(i) Were not directly related to pulmonary fibrosis or senescence;
(ii) Lacked mechanistic or translational relevance;
(iii) Consisted of conference abstracts, editorials, or non-peer-reviewed sources.

### 2.4. Study Selection and Data Synthesis

Studies were selected based on relevance to the topic and scientific quality. Titles and abstracts were initially screened, followed by full-text evaluation where appropriate. Given the narrative nature of this review, a formal systematic review framework (e.g., PRISMA) was not applied. Instead, emphasis was placed on conceptual relevance, methodological rigor, and the contribution of each study to understanding senescence-driven mechanisms in IPF.

A focused body of key mechanistic, translational, and clinically informative publications was prioritized for detailed conceptual synthesis, while additional supporting studies were integrated throughout the review to ensure balanced contextual coverage of the rapidly evolving IPF literature. Priority was given to:

- Recent studies (2015–2026);
- High-impact journals;
- Mechanistic and translational research;
- Studies with clinical relevance.

Data were qualitatively synthesized to identify major molecular pathways, including DNA damage response, telomere attrition, mitochondrial dysfunction, SASP signaling, and profibrotic pathways such as TGF-β.

Because this work was conducted as a narrative review rather than a formal systematic review, a standardized risk-of-bias assessment was not performed. Instead, emphasis was placed on methodological rigor, translational relevance, and scientific quality of included studies.

## 3. Results

### 3.1. Cellular Senescence in Idiopathic Pulmonary Fibrosis

Cellular senescence is increasingly regarded as a central feature of IPF pathogenesis. Accumulating evidence indicates that senescent cells are enriched in fibrotic lung tissue, where they contribute to disease persistence through dysregulated signaling and impaired regenerative capacity [6].

Alveolar type II (AT2) epithelial cells appear particularly vulnerable to senescence-associated alterations. In IPF-affected lungs, these cells demonstrate elevated expression of cell-cycle inhibitors such as p16INK4a and p21CIP1, alongside activation of DNA damage response pathways, consistent with stress-induced premature senescence [10,11]. Importantly, premature epithelial-cell senescence may occur independently of chronological aging and can be triggered by environmental exposure, oxidative injury, smoking, mitochondrial dysfunction, viral infection, and repeated alveolar microinjury. These mechanisms may contribute to fibrotic remodeling even in individuals without advanced biological aging. This functional decline in AT2 cells compromises epithelial repair mechanisms and promotes aberrant wound healing, thereby facilitating fibrotic remodeling [12].

Fibroblasts derived from patients with IPF also exhibit a senescent phenotype. These cells are characterized by resistance to apoptosis, sustained metabolic activity, and increased synthesis of extracellular matrix components [14,15]. Notably, senescent fibroblasts actively contribute to the maintenance of a profibrotic microenvironment by amplifying key fibrogenic signaling pathways [16].

Telomere shortening represents an additional and well-established driver of cellular senescence in IPF. Both familial and sporadic forms of the disease have been associated with mutations in telomerase-related genes, as well as accelerated telomere attrition. These findings support a mechanistic link between replicative senescence and the development of pulmonary fibrosis [17,18,19].

Experimental models have provided important insights into senescence-associated mechanisms in lung fibrosis. The bleomycin-induced fibrosis model remains one of the most widely used systems, enabling the study of epithelial injury, oxidative stress, and fibroblast activation. Data from murine studies suggest that the accumulation of senescent alveolar epithelial cells precedes the onset of fibrotic remodeling, reinforcing the concept that senescence is not merely a secondary consequence of tissue damage but an active contributor to disease progression [20,21,22]. This notion is further supported by evidence demonstrating that early senescence-related changes can predict subsequent fibrotic outcomes [21].

In addition, age-associated chronic inflammation, often referred to as “inflammaging,” creates a microenvironment that promotes both the induction and persistence of cellular senescence [12,23]. Persistent oxidative stress and mitochondrial dysfunction further reinforce these processes, forming self-sustaining signaling loops within fibrotic lung tissue [9,12].

Taken together, these findings highlight the central role of cellular senescence in driving epithelial dysfunction, fibroblast activation, and progressive, irreversible tissue remodeling in IPF.

### 3.2. Molecular Mechanisms of Senescence-Driven Fibrosis

Cellular senescence in IPF should be understood not as a passive consequence of aging, but as a dynamic, systems-level process sustained by persistent activation of the DNA damage response (DDR), telomere dysfunction, mitochondrial impairment, and profibrotic cytokine signaling. These processes operate within tightly interconnected feedback loops that stabilize epithelial dysfunction and perpetuate fibroblast activation [7,8,9,10,11].

#### 3.2.1. DNA Damage Response and Cell-Cycle Regulatory Networks

Persistent DDR activation represents one of the primary initiating mechanisms of epithelial senescence. Telomere erosion, oxidative DNA damage, and replication stress activate ATM/ATR kinases, leading to phosphorylation and stabilization of p53, followed by induction of p21CIP1 and irreversible cell-cycle arrest [9,10,24,25,26].

In parallel, the p16INK4a/Rb pathway reinforces this proliferative blockade through inhibition of CDK4/6 and maintenance of Rb in a hypophosphorylated state [24,27]. In IPF lung tissue, increased expression of p16INK4a and p21CIP1 has been consistently observed in alveolar epithelial cells located adjacent to fibroblast foci, indicating sustained DDR signaling in situ [10,11,12].

Beyond cell-cycle arrest, prolonged DDR activation induces chromatin remodeling and promotes the formation of senescence-associated heterochromatin foci (SAHF), thereby stabilizing transcriptional reprogramming toward a senescent state [24,25].

#### 3.2.2. Telomere Attrition as a Driver of Replicative Senescence

Telomere dysfunction represents a critical molecular link between aging and susceptibility to IPF. Germline mutations in telomerase components (TERT, TERC), together with accelerated telomere shortening, have been identified in both familial and sporadic cases of the disease [17,18].

Critically shortened telomeres are recognized as DNA double-strand breaks, triggering sustained p53 activation and replicative senescence. Functionally, this process reduces the regenerative capacity of AT2 epithelial cells, thereby impairing epithelial repair following injury [11,17].

Moreover, telomere dysfunction is closely associated with mitochondrial abnormalities, including increased production of reactive oxygen species (ROS) and metabolic instability, which further amplify DDR signaling in a feed-forward manner [7,28].

#### 3.2.3. Mitochondrial Dysfunction and Oxidative Stress Amplification

Mitochondrial dysfunction is a defining feature of senescent epithelial cells in IPF. Reduced expression of PINK1 and impaired mitophagy result in the accumulation of dysfunctional mitochondria, leading to excessive ROS production and disruption of cellular bioenergetics [29].

Elevated mitochondrial ROS not only exacerbate DNA damage but also activate redox-sensitive transcription factors, including NF-κB, which regulates the expression of SASP components [26,27]. In parallel, oxidative stress enhances TGF-β activation and promotes fibroblast differentiation [5,28].

Importantly, mitochondrial dysfunction and senescence form a self-reinforcing cycle: impaired mitophagy sustains ROS production, while senescence-associated metabolic reprogramming further suppresses mitochondrial turnover [29].

#### 3.2.4. TGF-β Signaling and Profibrotic Reprogramming

Transforming growth factor-β (TGF-β) plays a central role at the intersection of fibrosis and senescence. Canonical SMAD2/3 signaling induces transcription of extracellular matrix genes and drives myofibroblast differentiation [5].

At the same time, TGF-β directly promotes epithelial senescence through suppression of PTEN and activation of NF-κB signaling pathways [30]. Loss of PTEN leads to enhanced PI3K/AKT signaling, which supports the survival of senescent fibroblasts and contributes to their resistance to apoptosis [30].

Sustained TGF-β exposure therefore establishes a profibrotic microenvironment characterized by persistent accumulation of senescent cells and excessive matrix deposition [5,28]. Notably, senescent cells further amplify TGF-β signaling through SASP production, creating a self-perpetuating feedback loop linking epithelial dysfunction to fibroblast activation [10,28].

#### 3.2.5. mTOR, NF-κB, and SASP Regulation

The maintenance of the senescent phenotype requires continuous activation of metabolic and inflammatory signaling pathways. mTOR signaling enhances the translation of SASP components, while NF-κB functions as a key transcriptional regulator of pro-inflammatory cytokines, including IL-6, IL-8, and TNF-α [26,27].

Multiple upstream signals—such as DDR activation, mitochondrial ROS, and autocrine cytokine signaling—converge on NF-κB, transforming transient stress responses into chronic inflammatory signaling [26,31]. Persistent NF-κB activation stabilizes the SASP and contributes to sustained fibroblast activation in IPF [28].

#### 3.2.6. Autophagy and Proteostatic Imbalance

Autophagy serves as a critical homeostatic mechanism by removing damaged organelles and misfolded proteins. Under physiological conditions, it limits the accumulation of cellular stress and suppresses senescence.

In IPF, however, impaired autophagic flux accelerates senescence progression and exacerbates oxidative stress [12,29]. Experimental data suggest that restoration of mitophagy can attenuate fibrotic remodeling, highlighting its potential therapeutic relevance [29].

#### 3.2.7. Epigenetic Regulation of Senescence in IPF

Epigenetic mechanisms contribute to the stabilization of the senescent phenotype through persistent transcriptional reprogramming. The formation of SAHF alters chromatin accessibility and represses genes associated with cell proliferation [24].

In addition, DNA methylation changes and histone modifications promote long-term silencing of cell-cycle regulators while sustaining inflammatory gene expression [24,25]. Emerging evidence suggests that age-related epigenetic drift may predispose alveolar epithelial cells to maladaptive stress responses in IPF [7].

Thus, chromatin remodeling represents an additional regulatory layer linking environmental stressors to stable senescence programming.

The key molecular mechanisms contributing to senescence-driven fibrosis and their potential therapeutic targets are summarized in Table 1.

Taken together, these processes converge to sustain a persistent population of senescent cells, which in turn drives extracellular matrix accumulation and contributes to irreversible fibrotic remodeling.

**Figure 1:**
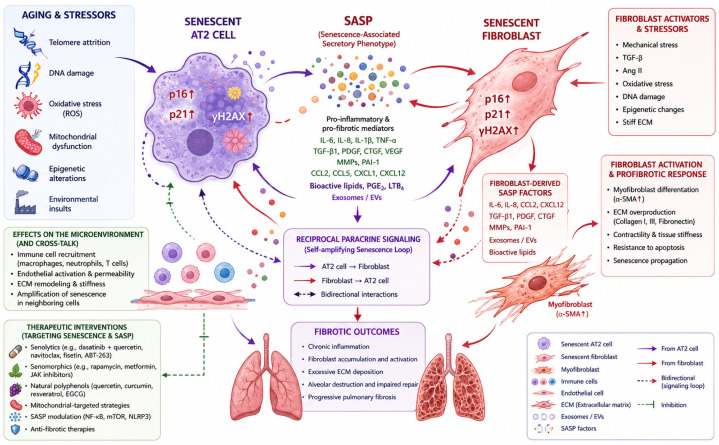
Molecular pathways linking cellular senescence to fibrotic remodeling in idiopathic pulmonary fibrosis. The vertical arrows indicate the direction of signaling interactions and progression of senescence-associated processes. Created by the authors based on current literature.

These molecular pathways operate in a highly interconnected manner, reinforcing both senescent cell persistence and the progressive deposition of extracellular matrix in IPF. A schematic overview of the key signaling pathways involved in senescence-associated fibrosis is presented in Figure 1.

Aging-related stressors, including telomere attrition, oxidative stress, mitochondrial dysfunction, and DNA damage, induce senescence in alveolar epithelial cells and fibroblasts. Senescent cells acquire a SASP characterized by secretion of IL-6, IL-8, TGF-β, and matrix metalloproteinases, which promote fibroblast activation, extracellular matrix deposition, chronic inflammation, and progressive fibrotic remodeling. Reciprocal signaling between senescent epithelial cells, fibroblasts, and SASP mediators creates self-reinforcing profibrotic feedback loops.

### 3.3. SASP and the Fibrotic Microenvironment

Cellular senescence is defined not only by irreversible growth arrest but also by the acquisition of a highly active and biologically influential secretory phenotype. SASP constitutes a context-dependent transcriptional program that includes pro-inflammatory cytokines, chemokines, growth factors, matrix metalloproteinases, and regulators of extracellular matrix turnover [5,13,26].

#### 3.3.1. Molecular Regulation of SASP

The induction and maintenance of the SASP are tightly controlled by interconnected signaling pathways, including persistent DNA damage response (DDR) activation, NF-κB signaling, and mTOR-dependent translational regulation [26,27]. Activation of ATM within the DDR pathway promotes NF-κB signaling through NEMO-dependent mechanisms, thereby initiating the transcription of pro-inflammatory mediators [26,27].

In parallel, mTOR signaling enhances the translation of IL-1α, a key upstream regulator of SASP amplification, establishing a self-sustaining inflammatory loop that reinforces senescent cell activity [27].

#### 3.3.2. Paracrine Senescence and Fibroblast Activation

SASP components, such as IL-6, IL-8, TGF-β, and matrix metalloproteinases, exert potent paracrine effects on neighboring cells, inducing secondary senescence through what is often referred to as the “bystander effect” [13,26]. This mechanism contributes to the spatial expansion of the senescent cell population and amplifies fibrotic signaling within the tissue microenvironment.

In particular, TGF-β and connective tissue growth factor (CTGF) secreted by senescent epithelial cells stimulate fibroblast proliferation and promote their differentiation into myofibroblasts, thereby enhancing extracellular matrix accumulation [5,28]. As a result, SASP-mediated signaling converts transient injury responses into persistent, self-sustaining tissue remodeling.

#### 3.3.3. Systemic SASP and Biomarker Potential

Circulating components of the SASP have been shown to correlate with disease severity and functional decline in IPF [22]. However, currently available markers, including p16INK4a expression and circulating cytokines, lack sufficient specificity, as they overlap with broader inflammatory processes.

Therefore, the identification of senescence-specific biomarker signatures remains a critical step toward the clinical implementation of senescence-targeted therapeutic strategies.

### 3.4. Therapeutic Strategies

Targeting cellular senescence has emerged as a promising approach aimed at modifying fundamental biological mechanisms that underlie the progression of IPF, rather than solely addressing downstream fibrotic manifestations. In a pilot first-in-human study, intermittent dasatinib plus quercetin administration was associated with improvements in physical performance measures, including six-minute walk distance and chair-stand performance, while demonstrating acceptable short-term tolerability. However, interpretation remains limited by the small sample size and exploratory study design.

#### 3.4.1. Senolytics

Senolytics are a class of pharmacological agents designed to selectively eliminate senescent cells by targeting their pro-survival pathways [20,21,24,27]. Preclinical and early translational studies have demonstrated that senolytic interventions can reduce senescent cell burden and improve functional outcomes in age-related conditions [25].

In experimental models of pulmonary fibrosis, the clearance of senescent cells has been associated with reduced extracellular matrix deposition and improved lung compliance [21,23]. Several natural and synthetic compounds, including procyanidin C1, the curcumin analog EF24, and resveratrol, have shown senolytic or SASP-modulating properties in preclinical settings [31,32,33,34,35].

Among the most extensively studied combinations is dasatinib plus quercetin, which targets key pro-survival signaling pathways, including BCL-2 family proteins and PI3K/AKT signaling, thereby promoting apoptosis in senescent cells [21,23]. Preclinical studies consistently report attenuation of fibrosis following treatment with this combination.

Importantly, a pilot clinical study evaluating dasatinib in combination with quercetin in patients with IPF demonstrated improvements in physical function alongside an acceptable safety profile, supporting the feasibility of senolytic approaches in humans [31]. While these findings require confirmation in larger randomized trials, they provide important proof-of-concept evidence for senescence-targeted therapy in fibrotic lung disease.

Beyond classical senolytics, alternative strategies include senomorphics—agents that suppress SASP activity without eliminating senescent cells. Targeting pathways such as mTOR, NF-κB, and JAK/STAT may attenuate profibrotic signaling while preserving potentially beneficial aspects of transient senescence responses [32,33].

#### 3.4.2. Natural Polyphenols as Senescence-Modulating Agents

Natural polyphenols have gained increasing attention as modulators of cellular senescence and fibrosis-related signaling pathways due to their antioxidant, anti-inflammatory, and epigenetic regulatory properties [31,33,34,35,36].

Quercetin is one of the most extensively studied flavonoids, demonstrating both senolytic and senomorphic effects, particularly in combination with dasatinib. Experimental studies indicate that this combination reduces senescent cell burden and attenuates fibrosis in bleomycin-induced lung injury models.

Curcumin and its synthetic analog EF24 have been shown to suppress oxidative stress–induced epithelial senescence and modulate PTEN-related signaling pathways. Similarly, resveratrol exerts protective effects through regulation of mitochondrial function and inflammatory signaling [33].

Other polyphenols, including hesperetin, luteoloside, and procyanidin C1, exhibit antifibrotic activity by restoring autophagic flux, reducing oxidative stress, and suppressing SASP-mediated fibroblast activation [31,32]. The molecular targets, mechanisms of action, and supporting experimental evidence for these compounds are summarized in Table 2.

While much of the current evidence remains at the preclinical stage, natural polyphenols are increasingly viewed as promising adjunctive agents capable of modulating senescence-associated pathways in idiopathic pulmonary fibrosis.

In parallel, emerging therapeutic approaches are exploring immune-mediated clearance of senescent cells, as well as targeted delivery systems designed to enhance tissue specificity and reduce off-target toxicity [33]. Additional emerging approaches include strategies aimed at restoring mitochondrial homeostasis and improving mitophagy in senescent epithelial cells. Extracellular vesicle-based therapies have recently attracted attention because of their potential to deliver anti-inflammatory, antioxidant, and mitochondrial-protective cargo directly to injured pulmonary tissue. Preclinical evidence suggests that extracellular vesicles derived from stem cells may attenuate oxidative stress, modulate SASP activity, and reduce fibroblast activation, although clinical validation remains limited. Despite these advances, several challenges continue to limit clinical translation, including the lack of reliable and specific biomarkers of senescence, the need for optimized dosing strategies, and the absence of long-term safety data.

**Figure 2:**
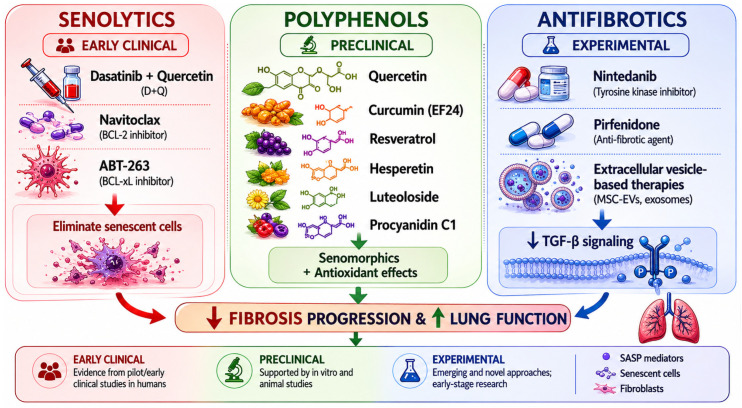
Therapeutic strategies targeting cellular senescence in idiopathic pulmonary fibrosis. Senolytics promote selective elimination of senescent cells, while senomorphics suppress SASP activity. Antifibrotic agents inhibit fibroblast activation and extracellular matrix deposition. Emerging approaches include immune-mediated clearance and targeted drug delivery systems to enhance therapeutic specificity. Therapeutic approaches are additionally categorized according to their translational stage, including preclinical, early clinical, and experimental strategies. The arrows indicate the direction of signaling interactions and therapeutic processes. Created by the authors based on current literature.

Taken together, these considerations suggest that targeting senescent cells represents a biologically grounded and potentially complementary strategy that could enhance the effectiveness of existing antifibrotic therapies in IPF (Figure 2).

### 3.5. Future Perspectives and Clinical Translation

Despite significant advances in elucidating the role of cellular senescence in idiopathic pulmonary fibrosis (IPF), several key challenges remain unresolved. In particular, the identification of reliable and disease-specific biomarkers of senescence continues to represent a major limitation for both research and clinical application [37,38,39,40,41,42,43,44,45]. Although markers such as p16INK4a, p21CIP1, and circulating SASP components are widely used, their specificity remains limited, as they often overlap with broader inflammatory and stress-related processes [34].

Emerging technologies, including single-cell transcriptomics and spatial omics, offer new opportunities to refine our understanding of senescent cell heterogeneity within fibrotic lung tissue. These approaches may enable the identification of distinct senescent subpopulations that differentially contribute to disease progression and may therefore represent more precise therapeutic targets.

Another critical area of development involves improving the selectivity and safety of senescence-targeted therapies. While senolytic agents have demonstrated encouraging results, their systemic administration raises concerns regarding potential disruption of physiological senescence processes that are essential for tissue repair and tumor suppression. Accordingly, future strategies should prioritize targeted delivery systems, optimized dosing regimens, and rational combination approaches with established antifibrotic therapies.

Translation into clinical practice will ultimately depend on the successful design of well-controlled randomized trials capable of evaluating long-term safety, functional respiratory outcomes, and survival benefits. In this context, a more nuanced understanding of the balance between transient, adaptive senescence and persistent, pathological senescence will be essential for achieving therapeutic efficacy [35].

Taken together, these considerations highlight that, although senescence-targeted approaches hold considerable promise, their clinical implementation remains complex. Bridging the gap between mechanistic insights and effective therapeutic strategies will be a key objective for future research in IPF.

## 4. Discussion

The integration of aging biology into fibrotic pathogenesis has fundamentally reframed IPF as a disorder of maladaptive tissue repair driven by persistent cellular senescence. While epithelial injury has long been recognized as a key initiating event [3,4], growing evidence indicates that senescence is not merely a downstream consequence but a central organizing mechanism coordinating chronic inflammation, fibroblast activation, and extracellular matrix deposition [10,11].

A major conceptual advance lies in understanding senescence as a network-level process sustained by interconnected signaling loops. Telomere attrition activates DNA damage response (DDR) pathways that compromise mitochondrial function; mitochondrial-derived reactive oxygen species stabilize NF-κB activation; SASP-associated cytokines enhance TGF-β signaling; and TGF-β, in turn, reinforces both senescence and fibroblast activation [5,7,28,30]. This tightly integrated signaling network provides a plausible mechanistic explanation for the persistence and apparent irreversibility of fibrotic remodeling, even after the initial injurious stimulus has been removed.

Despite these advances, several challenges continue to limit clinical translation. First, the marked heterogeneity of senescent cells complicates therapeutic targeting. Senescent cells do not represent a uniform population; depending on context, some subsets may retain beneficial roles in tissue repair and tumor suppression [27]. Consequently, non-selective elimination strategies may lead to unintended effects.

Second, widely used experimental models—particularly bleomycin-induced fibrosis—do not fully recapitulate the chronic and progressive nature of human IPF [21]. This limitation highlights the need for more representative translational models that incorporate aging-related susceptibility and long-term disease dynamics.

Third, the lack of reliable biomarkers for senescent cell burden remains a critical barrier. Composite molecular signatures integrating p16INK4a expression, SASP profiles, and epigenetic markers may provide more accurate tools for patient stratification and treatment monitoring [34,37,40].

Important safety considerations remain unresolved for senescence-targeted therapies. Systemic elimination of senescent cells may interfere with physiological senescence processes involved in tissue repair, wound healing, and tumor suppression. In addition, off-target toxicity, limited tissue specificity, and the absence of long-term safety data remain major barriers to clinical translation. Future therapeutic strategies will likely require selective targeting of pathogenic senescent-cell subpopulations while preserving adaptive transient senescence responses.

Taken together, these considerations suggest that effective clinical translation will require selective targeting of pathogenic senescent cell subpopulations while preserving transient, physiological senescence responses. In this context, systems-level approaches integrating genomic instability, mitochondrial signaling, inflammatory regulation, and epigenetic remodeling may offer a framework for the development of precision senotherapeutics.

## 5. Conclusions

Cellular senescence is strongly associated with fibrotic progression and may contribute to disease persistence as a central pathogenic mechanism linking aging to idiopathic pulmonary fibrosis. The accumulation of senescent epithelial cells and fibroblasts, together with sustained activation of DNA damage and stress-response pathways and persistent SASP signaling, drives fibroblast activation and excessive extracellular matrix deposition.

Advances in understanding key mechanisms—including mitochondrial dysfunction, telomere attrition, dysregulated TGF-β signaling, and impaired autophagy—have significantly expanded our knowledge of senescence-driven fibrosis. Importantly, emerging preclinical and early clinical evidence suggests that targeting senescent cells may influence fundamental disease processes rather than merely slowing disease progression.

Future efforts should focus on the development of robust senescence biomarkers, optimization of senescence-targeted therapeutic strategies, and the implementation of well-designed clinical trials. Overall, targeting cellular senescence represents a biologically grounded and translationally promising approach for the treatment of IPF [20,21,22,23,24,25,26,27,28,29,30,31,32,33,34,35,36,37,38,39,40,41,42,43,44,45].

## 6. Limitations

This review has several limitations that should be acknowledged. First, the manuscript was designed as a narrative review rather than a formal systematic review or meta-analysis; therefore, PRISMA methodology, quantitative synthesis, and standardized risk-of-bias assessment were not performed. Second, the included studies demonstrate substantial heterogeneity in experimental design, translational models, and clinical populations, which may limit direct comparability of findings. Third, although emphasis was placed on high-impact and mechanistically informative studies, selective bias cannot be fully excluded.

In addition, commonly used experimental models, including bleomycin-induced fibrosis, do not fully reproduce the chronic and progressive course of human IPF. This limitation may restrict the direct translation of preclinical findings into clinical practice.

## Figures and Tables

**Table 1:** Key molecular mechanisms of cellular senescence in idiopathic pulmonary fibrosis and potential therapeutic targets.

Senescence-Associated Mechanism	Key Molecular Mediators	Role in IPF	Therapeutic Strategy
Telomere attrition	TERT, TERC, p53	Replicative senescence of AT2 cells	Telomerase activation
DNA damage response	p53, p21, ATM/ATR	Cell-cycle arrest	DDR modulation
Mitochondrial dysfunction	ROS, PGC-1α	Oxidative stress, apoptosis resistance	Antioxidants, mitophagy enhancers
TGF-β signaling	TGF-β1, SMAD2/3	Fibroblast activation	TGF-β inhibitors
SASP	IL-6, IL-8, TNF-α, MMPs	Chronic inflammation	Senomorphics
Anti-apoptotic pathways	BCL-2 family	Survival of senescent cells	Senolytics

Source: Compiled by the authors based on data reported in references [10,11,12,13,17,24,25,26,27,28,29,30].

**Table 2:** Natural polyphenols as modulators of cellular senescence and pulmonary fibrosis.

Compound	Targets	Mechanism	Evidence
Quercetin	PI3K/AKT, BCL-2	Senolytic, SASP suppression	Clinical + preclinical
Dasatinib + Quercetin	Tyrosine kinases	Senolytic	Pilot clinical data
Procyanidin C1	BCL-2	Senescent cell clearance	Animal models
Curcumin	NF-κB	Anti-inflammatory	Experimental
EF24	PTEN/PI3K	Anti-senescence	Experimental
Resveratrol	SIRT1, NF-κB	Mitochondrial protection	Preclinical
Hesperetin	Autophagy	ROS reduction	Experimental

Source: Compiled by the authors based on data reported in references [21,23,31,32,33,34,35,36]. ROS: Reactive Oxygen Species; SASP: Senescence-Associated Secretory Phenotype.

## Data Availability

No new data were created or analyzed in this study.

## Supplementary Materials

The following supporting information can be downloaded at: https://www.mdpi.com/article/10.3390/diseases14060201/s1. Figure S1. Flow diagram illustrating the literature search and study selection process used for this narrative review. Records were identified through searches of PubMed, Scopus, and Web of Science databases. Following title and abstract screening, full-text articles were assessed for eligibility, and studies meeting the predefined inclusion criteria were incorporated into the qualitative narrative synthesis.

## Abbreviations

The following abbreviations are used in this manuscript:

IPF	Idiopathic Pulmonary Fibrosis
SASP	Senescence-Associated Secretory Phenotype
AT2	Alveolar Type II
TGF-β	Transforming Growth Factor Beta
ROS	Reactive Oxygen Species
ECM	Extracellular Matrix
