# Supplementary figures and images for: Cellular Senescence in Idiopathic Pulmonary Fibrosis: Molecular Mechanisms, Pathogenic Networks, and Emerging Therapeutic Targets
Source: Diseases. 2026 Jun 4;14(6):201. doi: 10.3390/diseases14060201 (PMC13298089; doi:10.3390/diseases14060201)

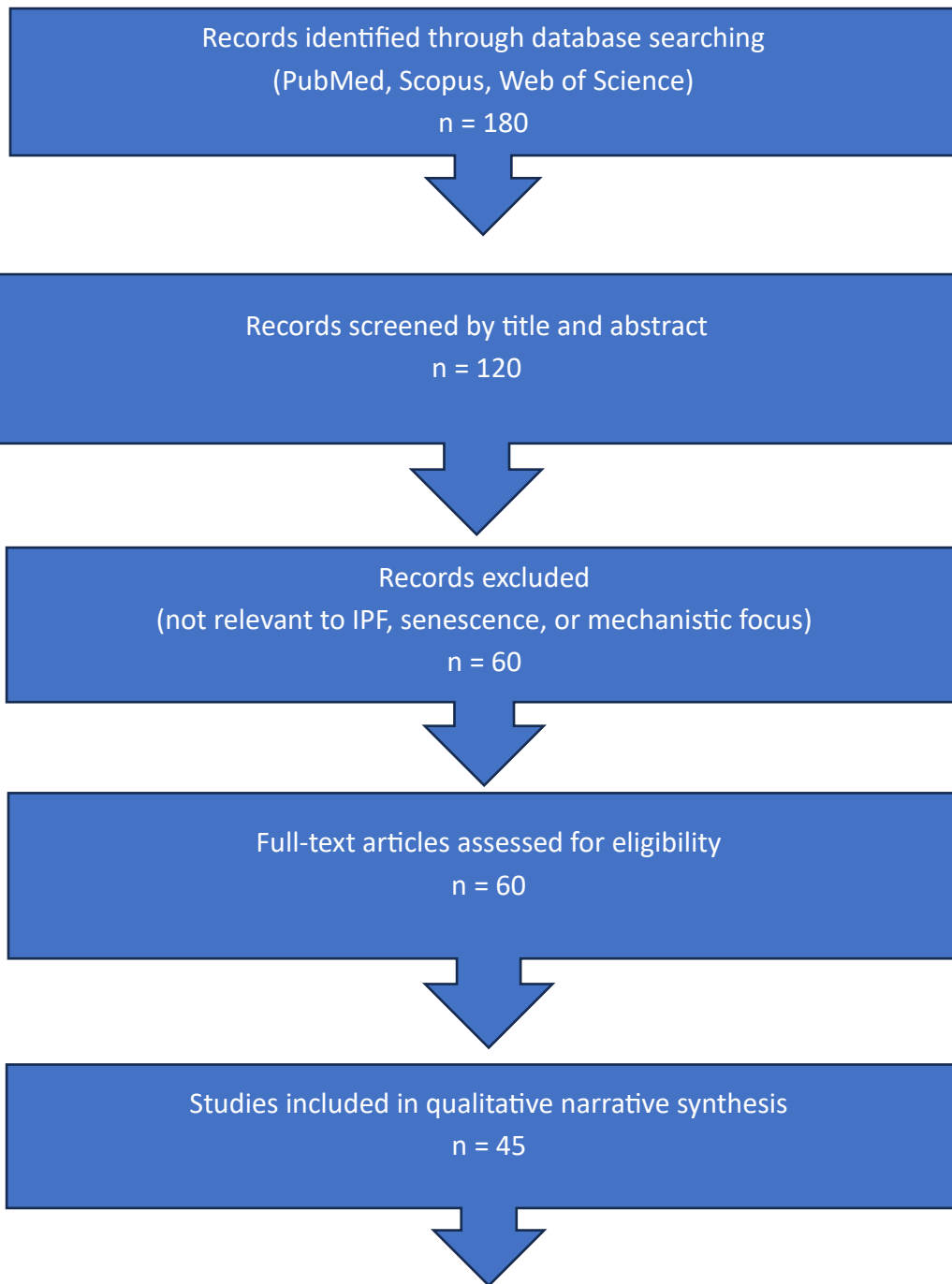

Supplement: Supplementary file 1 [file diseases-14-00201-s001.zip › diseases-4315439-supplementary-1.pdf]
